# Abdominal Muscles and Metabolic Syndrome According to Patient Sex: A Retrospective Cross-Sectional Study

**DOI:** 10.3390/healthcare9091197

**Published:** 2021-09-10

**Authors:** Tae Young Lee, Young-Jee Jeon, Chung Reen Kim, Byung Ju Kang, Gyung-Min Park

**Affiliations:** 1 Department of Radiology, Ulsan University Hospital, University of Ulsan College of Medicine, Ulsan 44033, Korea; 0734147@uuh.ulsan.kr; 2 Department of Family Medicine, Ulsan University Hospital, University of Ulsan College of Medicine, Ulsan 44033, Korea; 3 Department of Physical Medicine and Rehabilitation, Ulsan University Hospital, University of Ulsan College of Medicine, Ulsan 44033, Korea; 4 Department of Internal Medicine, Ulsan University Hospital, University of Ulsan College of Medicine, Ulsan 44033, Korea; juriver@uuh.ulsan.kr (B.J.K.); gmpark@uuh.ulsan.kr (G.-M.P.)

**Keywords:** metabolic syndrome, computed tomography, attenuation, abdominal muscle, sex characteristics

## Abstract

Computed tomography (CT) is a reference method for measuring skeletal muscle mass, and the amount of fat in the skeletal muscle can be calculated based on CT attenuation. This study aimed to comprehensively investigate the effect of muscle quality and quantity on metabolic syndrome (MetS) according to sex. This retrospective cross-sectional study enrolled 8081 individuals aged ≥20 years who underwent self-referral abdominopelvic CT at our hospital. The total abdominal muscle area (TAMA), low-attenuation abdominal muscle area (LAMA), normal-attenuation abdominal muscle area (NAMA), and extramyocellular lipid area (EMCLA) were measured using cross-sectional CT data of the L3 lumbar vertebrae. The TAMA and NAMA showed negative correlations with risk factors for MetS and a positive correlation with high-density lipoprotein cholesterol, whereas the LAMA and EMCLA showed an inverse trend in both the sexes (*p* < 0.001). After adjusting for various factors, a higher LAMA index and the ratio of LAMA to TAMA were associated with a higher prevalence of MetS. High TAMA indices were associated with a lower prevalence of MetS. Furthermore, muscle quality and quantity were associated with the prevalence of MetS in both males and females. However, the LAMA showed a stronger association with MetS in males than in females.

## 1. Introduction

Fat that cannot be stored in adipose tissue is stored in lean tissues, where it is called ectopic fat. Ectopic fat accumulation in skeletal muscle is related to insulin resistance [1] and it is classified into intramyocellular lipids (IMCLs) and extramyocellular lipids (EMCLs) according to the location of the muscle and fat. Fat located between or within muscles that can be visualized using computed tomography (CT) is considered to be EMCL (or intermuscular adipose tissue), and lipid droplets accumulated in muscle cells are considered to be IMCLs [2]. IMCLs are associated with insulin resistance, dyslipidemia, and metabolic syndrome (MetS) [3,4]. A previous study has shown that the effect of IMCL on cardiometabolic risk differs according to sex [5].

In glycemic regulation, skeletal muscles play important roles in glucose storage and consumption and extract approximately one-third of the glucose load [6]. In previous studies, a higher skeletal muscle mass (SMM) has been reported to lower the risk of MetS. Additionally, in abdominal obesity, the risk of MetS significantly increases as SMM decreases [7]. In a meta-analysis on sarcopenia, the risk of MetS increased by two-fold in non-obese patients with sarcopenia compared with those without sarcopenia [8]. However, another study reported contradictory results, i.e., larger muscle mass was inversely related to insulin sensitivity [9]. These conflicting results suggest that prediction of the risk of MetS based on SMM alone can be misleading.

The authors hypothesized that some muscle types lower the risk of MetS, whereas others increase it and that the amount of IMCLs may influence this risk. Most previous studies have measured muscle and fat mass using dual X-ray absorptiometry (DEXA) [10,11,12] or bioelectrical impedance analysis (BIA) [13,14]. These methods can easily measure the amount of subcutaneous or visceral fat but not that of IMCL. A direct muscle biopsy has been established as the gold standard for measuring fat content in muscles; however, it is an invasive method. In recent years, CT has been gaining popularity as a noninvasive method for measuring fat content in muscles. CT measurements were reported to significantly correlate with biopsy measurements [15]. In CT, low-attenuation muscle indicates a fat-rich area [16] with a higher triglycerides content; triglycerides are the main components of muscle fat [17].

Only few studies have reported the relationship between IMCL and MetS using CT. In some previous studies, only low-attenuation muscles in MetS were analyzed without any comparative analysis [18,19], and in another study, the analyses involved comparisons of low-attenuation and normal-attenuation muscles but did not adjust for comorbidities [20]. In these studies, the number of female participants with MetS was lower than that of males; therefore, it was difficult to determine the above-mentioned associations in females. To address this gap in knowledge, our study aimed to investigate the effect of different amounts of fat in low-attenuation and normal-attenuation muscles, as classified based on CT, on MetS in a healthy population after adjusting for traditional risk factors, such as visceral fat and IMCL, according to sex.

## 2. Materials and Methods

### 2.1. Study Participants

**Figure 1:**
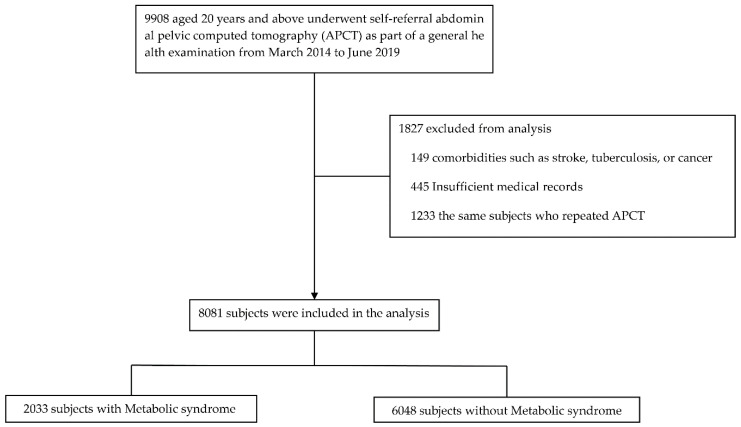
Overview of the study population.

We retrospectively obtained data from 9908 participants aged ≥20 years who underwent self-referral abdominopelvic CT (APCT) evaluation as a part of routine check-ups at the Health Promotion Center, Ulsan University Hospital, between March 2014 and June 2019. Ulsan is a representative industrial city in Korea with many large companies, including shipbuilding and automobile companies. These companies provide health check-ups every 2 years for the welfare and health of their employees. The exclusion criteria were as follows: (1) presence of chronic diseases affecting muscle mass, such as stroke, tuberculosis, or cancer; (2) insufficient medical records; and (3) having undergone multiple APCT during the study period. Finally, 8081 individuals were enrolled in the study (Figure 1). Clinical and laboratory variables were collected using the clinical data warehouse platform in conjunction with electronic medical records at the Ulsan University Hospital. This study was approved by the local Institutional Review Board of the Ulsan University Hospital, Ulsan, Korea (No. 2020-07-028-002); it conformed to the principles outlined in the Declaration of Helsinki. The need for informed consent was waived owing to the retrospective nature and the anonymization of the data included in the study.

### 2.2. Clinical and Laboratory Measurements

Data on clinical and lifestyle factors (e.g., comorbidities, such as hypertension, diabetes, dyslipidemia, and cardiovascular disease; smoking status; alcohol consumption; physical activity; and menopausal status) were collected from systemized self-reported questionnaires issued to the participants prior to their check-up. Excessive alcohol consumption was defined as consuming more than 14 drinks/week for males and 7 drinks/week for females, according to the National Institute on Alcohol Abuse and Alcoholism guidelines [21]. Performing more than 150 min/week of moderate-intensity activity or 75 min/week of vigorous activity or a combination of both was considered as moderate-to-vigorous physical activity, according to the American College of Sports Medicine guidelines [22]. Participants who reported no exercise activity were classified into the sedentary group, whereas those with an exercise routine between sedentary and moderate-to-vigorous physical activity were classified into the light activity group.

Height and weight were obtained while the participants wore light clothing and had no shoes on. Body mass index (BMI) was calculated as the weight (kg) divided by the square of the height (m^2^). Waist circumference (cm) was measured midway between the costal margin and the iliac crest at the end of a normal expiration. The classification of normal weight, overweight, and obese based on BMI was in accordance with the World Health Organization guidelines in the Asia-Pacific region: normal weight (BMI < 23 kg/m^2^), overweight (BMI ≥ 23 and <25 kg/m^2^), and obese (BMI ≥ 25 kg/m^2^) [23]. Blood pressure was measured on the right arm after a ≥5 min rest using an automatic manometer with an appropriate cuff size. After overnight fasting, early morning blood samples were collected from the antecubital vein into vacuum tubes and subsequently analyzed at the central certified laboratory of the Ulsan University Hospital. The concentrations of fasting blood glucose, high-density lipoprotein (HDL) cholesterol, low-density lipoprotein cholesterol, and triglycerides were measured.

MetS was defined based on the revised National Cholesterol Education Program criteria proposed by the American Heart Association/National Heart, Lung, and Blood Institute [24]. MetS requires the presence of at least three of the following five components: (1) abdominal obesity (waist circumference ≥90 cm for Asian men and ≥80 cm for Asian women), (2) triglyceride level ≥150 mg/dL (3) HDL cholesterol level <40 mg/dL for males or <50 mg/dL for females or those receiving drug treatment, (4) systolic/diastolic blood pressure ≥130/85 mmHg or receiving drug treatment, and (5) fasting plasma glucose concentration ≥100 mg/dL or receiving drug treatment.

### 2.3. APCT Image Acquisition and Analysis

All CT images were obtained using the SOMATOM Definition Flash system (Siemens Healthcare, Erlangen, Germany). For contrast enhancement, 100–120 mL of 150 mg I/mL iopromide (Xenetix 350; Guerbet, Roissy, France) was administered intravenously at a rate of 3–4 mL/s using an automatic power injector through an 18-gauge intravenous cubital line, followed by a 20 mL saline flush at the same flow rate. Enhanced images were obtained after a fixed 80 s delay after contrast injection. The scanning parameters were as follows: beam collimation, 128 × 0.6 mm; beam pitch, 0.6; gantry rotation time, 0.5 s; field of view to fit, 100 kVp. An automatic exposure control system (CARE Dose 4D, Siemens Medical Solutions, Erlangen, Germany) was used. Post contrast-enhanced CT scans were reconstructed using a kernel (I40f) and a slice thickness of 3 mm.

**Figure 2:**
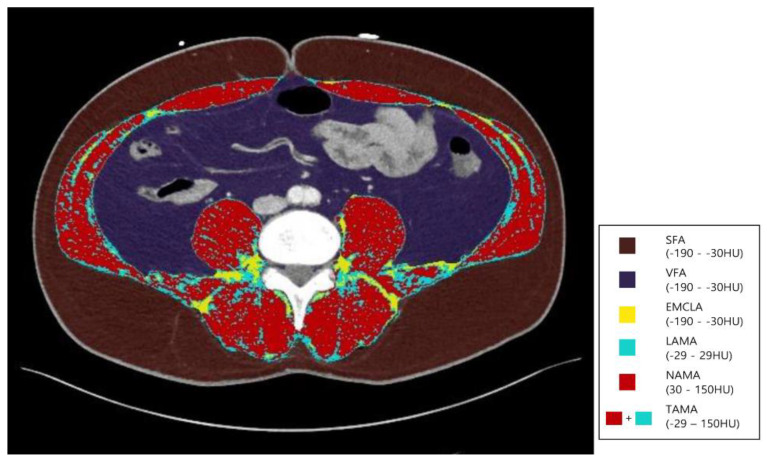
Segmental abdominal body fat and muscle analysis at the L3 vertebra on abdominopelvic computed tomography. HU, Hounsfield units; IMAT, intermuscular adipose tissue; LAMA, low-attenuation abdominal muscle area; NAMA, normal-attenuation muscle area; SFA, subcutaneous fat area; TAMA, total abdominal muscle area; VFA, visceral fat area.

Body composition was evaluated with APCT using the Asan-J software, which was developed based on ImageJ (NIH, Bethesda, MD, USA) [25,26]. Two consecutive axial CT images leveled at the inferior endplate of the L3 lumbar vertebra were captured and then averaged for each patient. Using the Asan-J software, we calculated the total abdominal muscle area (TAMA) (cm^2^), including all muscles in the field (psoas, paraspinal, transversus abdominis, rectus abdominis, quadratus lumborum, and internal/external obliques), with predetermined Hounsfield unit (HU) thresholds on CT. The TAMA was divided into a low-attenuation abdominal muscle area (LAMA) and a normal-attenuation abdominal muscle area (NAMA) based on HUs on CT (TAMA, −29 to 150 HU; LAMA, −29 to 29 HU; NAMA, 30 to 150 HU). The EMCL area was determined using HU thresholds (−190 to −30 HU) on CT in the muscle field. The TAMA and LAMA did not comprise an intermuscular fat area. Furthermore, the visceral fat area (VFA) (cm^2^) and the subcutaneous fat area (SFA) (cm^2^) were outlined and evaluated using adipose tissue thresholds on CT (−190 to −30 HU) (Figure 2). These areas were adjusted for BMI by dividing the muscle area by the participant’s BMI; these were termed the TAMA index (TAMAI) (TAMAI = TAMA [cm^2^]/BMI [kg/m^2^]), LAMA index (LAMAI) (LAMAI = LAMA [cm^2^]/BMI [kg/m^2^]), NAMA index (NAMAI) (NAMAI = NAMA [cm^2^]/BMI [kg/m^2^]), EMCLA index (EMCLAI) (EMCLAI = EMCLA [cm^2^]/BMI [kg/m^2^]), VFA index (VFAI) (VFAI = VFA [cm^2^]/BMI [kg/m^2^]), and SFA index (SFAI) (SFAI = SFA [cm^2^]/BMI [kg/m^2^]).

### 2.4. Statistical Analysis

The clinical and metabolic characteristics are presented as frequencies with percentages for categorical variables and as means with standard deviations for continuous variables. Between-group comparisons were performed using Pearson’s chi-square test for categorical variables and Student’s *t*-test for numerical variables. Pearson’s correlation coefficient was calculated to determine the correlation between metabolic risk factors and CT indices. Additionally, multivariate logistic regression analyses were performed to evaluate the independent relationships between MetS and the abdominal muscle and fat area. The covariates used in the multivariate analysis were selected based on previous studies [20,27,28] and were analyzed separately for males and females. All statistical analyses were performed using the Statistical Package for the Social Sciences version 24 for Windows (SPSS, IBM Corporation, Armonk, NY, USA). A *p*-value < 0.05 was considered significant for all analyses.

## 3. Results

### 3.1. Participant Characteristics

The mean age of the study participants was 52.8 ± 9.4 years, and 4835 (59.8%) participants were males. Among the study participants, 2033 (25.2%) had MetS. The baseline characteristics of the study population are listed in Table 1. Participants with MetS were older (*p* = 0.03 for males; *p* < 0.01 for females), more obese, and had more comorbidities (*p* < 0.01). In addition, a higher proportion of male participants with MetS were current smokers (*p* < 0.01) and had sedentary lifestyles (*p* < 0.01). Both male and female participants with MetS consumed high amounts of alcohol (*p* < 0.01).

### 3.2. CT Findings

Table 2 shows the CT findings according to MetS. The mean SFA, VFA, and EMCLA in the study participants were 143.7 ± 60.1 cm^2^, 104.8 ± 61.4 cm^2^, and 5.5 ± 4.0 cm^2^, respectively. In addition, the mean TAMA, NAMA, and LAMA were 137.0 ± 34.4 cm^2^, 112.1 ± 32.0 cm^2^, and 24.9 ± 10.5 cm^2^, respectively.

The SFA, VFA, EMCLA, TAMA, NAMA, and LAMA were higher in male participants with MetS than in those without MetS (*p* < 0.01). In females, only the NAMA was lower in female participants with MetS than in those without MetS (*p* = 0.009); other muscle areas were similar between females and males (*p* < 0.01). However, the TAMAI and NAMAI were lower in participants with MetS than in those without MetS in both male and female participants (*p* < 0.01). The LAMAI, SFAI, VFAI, EMCLAI, and percentage ratio of LAMA to TAMA were higher in participants with MetS in both the sexes (*p* < 0.01).

In sex-related comparisons, the SFA, EMCLA, SFAI, EMCLAI, and ratio of LAMA to TAMA were higher in females with MetS than in males with MetS (EMCLA: *p* = 0.023; the other parameters: *p* < 0.01). Furthermore, the VFA, TAMA, NAMA, LAMA, VFAI, TAMAI, NAMAI, and LAMAI were higher in males with MetS than in females with MetS (*p* < 0.01).

### 3.3. Correlation between Metabolic Risk Factors and the TAMAI, NAMAI, LAMAI, SFAI, VFAI, and EMCLAI

We investigated the correlation between the risk factors of MetS and the TAMAI, NAMAI, LAMAI, SFAI, VFAI, and EMCLAI. The TAMAI and NAMAI showed negative correlations with waist circumference, triglycerides, fasting blood glucose, diastolic blood pressure, and systolic blood pressure and a positive correlation with HDL cholesterol, whereas the LAMAI and SFAI, VFAI, and EMCLAI showed an inverse pattern, i.e., positive correlations with waist circumference, triglycerides, fasting blood glucose, diastolic blood pressure, and systolic blood pressure and a negative correlation with HDL cholesterol. All correlation coefficient values were significant (*p* < 0.01) (Table 3).

### 3.4. Association between MetS and Abdominal Muscles and Fat Area

After adjusting for age, comorbidities, smoking status, alcohol consumption, physical activity, visceral fat index, and EMCL index, multivariable logistic regression analysis showed that the TAMAI (males: odds ratio [OR], 0.937; 95% confidence interval [CI], 0.835–1.052; *p* = 0.271; postmenopausal females: OR, 0.820; 95% CI, 0.658–1.022; *p* = 0.077; and premenopausal females: OR, 0.546; 95% CI, 0.352–0.941; *p* = 0.028) was significantly associated with a lower prevalence of MetS only in premenopausal females. The NAMAI (males: OR, 0.836; 95% CI, 0.743–0.940; *p* = 0.003; postmenopausal females: OR, 0.754; 95% CI, 0.601–0.946; *p* = 0.015; and premenopausal females: OR, 0.534; 95% CI, 0.321–0.887; *p* = 0.015) was significantly associated with a lower prevalence of MetS. Moreover, the LAMAI (males: OR, 1.771; 95% CI, 1.359–2.308; *p* < 0.01; postmenopausal females: OR, 1.402; 95% CI, 0.861–2.284; *p* = 0.174; and premenopausal females: OR, 1.420; 95% CI, 0.350–5.765; *p* = 0.623) was significantly associated with a higher prevalence of MetS only in males, and the ratio of LAMA to TAMA (males: OR, 1.040; 95% CI, 1.023–1.058; *p* < 0.01; postmenopausal females: OR, 1.025; 95% CI, 1.004–1.046; *p* = 0.022; and premenopausal females: OR, 1.050; 95% CI, 0.987–1.117; *p* = 0.124) was significantly associated with the prevalence of MetS in males and postmenopausal females (Table 4).

To examine the association between sole MetS and abdominal muscle and fat area, we reanalyzed multivariable logistic regression after excluding participants with comorbidities (hypertension, diabetes, dyslipidemia, and cardiovascular disease). After excluding comorbidities, the NAMAI was significantly associated with a lower prevalence of MetS in males (OR, 0.784; 95% CI, 0.651–0.946 *p* = 0.011). The ratio of LAMA to TAMA was significantly associated with the prevalence of MetS in males (OR, 1.032; 95% CI, 1.002–1.063 *p* = 0.034). There were no significant variables in premenopausal females and postmenopausal females (Table A1 in Appendix A).

## 4. Discussion

In this study, we classified the abdominal muscle into the LAMA and NAMA according to CT attenuation. After adjusting for well-known and relevant risk factors of MetS, we found that a higher NAMAI was associated with a lower prevalence of MetS. However, a higher LAMAI was associated with a higher prevalence of MetS in males but not with a higher prevalence of MetS in females. The ratio of LAMA to TAMA was significantly associated with a higher prevalence of MetS in males and postmenopausal females but not in premenopausal females. Therefore, our study suggests that both quantitative and qualitative features of the abdominal skeletal muscle are important for determining the association with a prevalence of MetS, and this association differs according to sex.

Most previous studies investigating the association between MetS and SMM by dual DEXA [10,11,12] or BIA [13,14] revealed that an increased SMM reduces the risk of MetS, thus highlighting the positive effects of skeletal muscle on insulin control. Skeletal muscle is a major site for excess fat storage. Thus, even without an increase in pure muscle fibers, an increase in IMCL can increase the skeletal muscle volume. Excess visceral adiposity induces ectopic fat deposition in the liver, heart, and skeletal muscles and results in abdominal obesity. Abdominal obesity is a key component of MetS-associated insulin resistance [29]. Fat infiltration in the skeletal muscle releases pro-inflammatory cytokines in close proximity to muscle fibers and increases the rate of lipolysis within the skeletal muscle. The relationship between MetS and IMCL deposition has been addressed in previous studies. In particular, lipid accumulation in abdominal muscles is positively associated with insulin resistance [30], thereby increasing the risk of developing type 2 diabetes [31]. Therefore, both muscular quantity and quality should be considered.

As mentioned earlier, DEXA and BIA can only measure the total SMM but not the degree of lipid accumulation. Direct muscle biopsy, although useful in measuring the exact amount of IMCL, is invasive. In contrast, CT is a noninvasive and reliable method [32] that has been gaining popularity [16,18]. In CT, the decrease in skeletal muscle attenuation may result from an increased intramuscular lipid amount and increased glycogen and water concentrations beyond the physiological ranges. Such an increase in glycogen and water concentrations is unusual; therefore, reduced muscle attenuation in CT is mainly associated with intramyocellular lipids [33]. In previous studies on MetS, low attenuation in the thigh [16,18] and abdominal muscles [18,19] measured by CT was associated with insulin resistance; this association was stronger for abdominal muscles [19].

The Framingham Heart Study with a large cohort identified associations between paraspinal muscle attenuation and metabolic risk factors [34] and found that reduced muscle attenuation in males was not significantly associated with the risks of insulin resistance and MetS. However, that study had some limitations regarding the analysis of the effects of muscle attenuation. First, unlike our study, where the attenuation was divided into LAMA and NAMA, the Framingham Heart Study only used the LAMA. Second, these results were not adjusted for BMI. Adjusting the degree of obesity with BMI is important when considering the correlation between IMCL and visceral fat [34]. For example, a study on the relationship between sarcopenic obesity and MetS showed different results depending on the adjustment method. When muscle mass was corrected with height, muscle mass, and MetS, a positive association was revealed, whereas when muscle mass was corrected with BMI, a negative association was revealed [10].

One of the strengths of our study is that we derived results by adjusting for various relevant risk factors of MetS to determine the independent effects of IMCLs on MetS. The results showed that IMCLs were independently related to MetS. However, previous studies have reported different results. In a study including 808 Japanese participants, APCT was used to measure the skeletal mass index (SMI) and skeletal mass attenuation (SMA); the participants were divided into four groups based on the median of the total SMI and SMA [20]. The odds for the prevalence of MetS in the low-SMI and -SMA group were 5.86 for males and 7.32 for females (reference: high-SMI and -SMA group). Therefore, both SMI and SMA seemed to be independently associated with the number of MetS components in both males and females. However, after adjusting for visceral fat, the association was insignificant in males. In our study, the effect of IMCLs was independently correlated with MetS even after adjusting for visceral fat and EMCL. This difference might have been influenced by a small number of study participants in the previous study [20], where the number of female patients with MetS was small (*n* = 22), making it challenging to analyze in detail. In the present study, we included 737 females with MetS; this allowed us to examine the effect of IMCLs on MetS in females. Furthermore, we excluded participants with chronic diseases such as stroke, malignant tumors, and tuberculosis that cause muscle loss and adjusted for comorbid diseases, such as hypertension, diabetes, dyslipidemia, and cardiovascular disease. Therefore, our study could determine the effects of IMCLs independently.

In our study, the NAMA lowered the risk of MetS in both males and females, but the effect of the LAMA differed according to sex. In males, the LAMA increased the risk of MetS, but there was no significant effect on females. Interestingly, the ratio of LAMA to TAMA increased the risk of MetS in males and postmenopausal women but not in premenopausal women. Accumulation sites of ectopic fat differ according to sex, being mainly in the liver in males and in the skeletal muscle in females [1]. In addition, body composition, which increased the cardiometabolic risk, differed according to sex; visceral fat in women and IMCLs in men had more negative effects; the latter effect was because IMCLs were associated with higher inflammatory markers in males but not in females [5]. This could be a potential mechanism explaining why LAMA was not associated with MetS in females in this study. Sex hormones, especially low estrogen levels, can explain the different accumulation sites of ectopic fat between sexes and the different body composition effects on cardiometabolic risk [5]. The ratio of LAMA to TAMA in this study significantly increased the prevalence of MetS in postmenopausal females and in males but not in premenopausal females, which could also be explained by the effects of sex hormones.

Several limitations should be considered in the interpretation of our study. First, this study was a cross-sectional study; therefore, causal relationships cannot be interpreted. Second, a selection bias might have occurred because the study recruited voluntary participants who underwent APCT as a part of their routine check-ups. However, this study was conducted with a relatively large number of individuals among the general population. Third, we did not evaluate the functional parameters of muscle strength, such as a handgrip or gait speed. Last, we did not measure the direct IMCL amount. The LAMA is assumed to have increased levels of IMCL accumulation in the muscle, whereas the NAMA is assumed to have relatively decreased levels of IMCL accumulation [33].

## 5. Conclusions

In this large cross-sectional study on individuals who underwent CT, both quantitative and qualitative features of the muscle were significantly associated with MetS. In addition, these associations were different between males and females. Therefore, we believe that this study provides a new perspective on the relationship between abdominal muscles and MetS according to sex. However, these findings need to be further investigated and validated in future studies.

## Figures and Tables

**Table 1:** Characteristics of the study participants (*n* = 8081).

Variables	Total	Male (*n* = 4835)			Female (*n* = 3246)		
		MetS (*n* = 1296)	No MetS (*n* = 3539)	*p*-Value	MetS (*n* = 737)	No MetS (*n* = 2509)	*p*-Value
Age, year	52.8 ± 9.4	53.1 ± 8.9	52.2 ± 9.5	0.03	58.6 ± 8.5	51.9 ± 9.1	<0.01
BMI, kg/m^2^	24.0 ± 3.1	26.5 ± 2.8	23.9 ± 2.6	<0.01	25.4 ± 2.9	22.3 ± 2.7	<0.01
				<0.01			<0.01
Normal (BMI < 23 kg/m^2^)	3097 (38.3)	110 (8.5)	1261 (35.6)		146 (19.8)	1580 (63.0)	
Overweight (BMI ≥ 23 and <25 kg/m^2^)	2206 (27.3)	260 (20.1)	1200 (33.9)		208 (28.2)	538 (21.4)	
Obese (BMI ≥ 25 kg/m^2^)	2778 (34.4)	926 (71.5)	1078 (30.5)		383 (52.0)	391 (15.6)	
Waist circumference, cm	84.9 ± 8.2	92.6 ± 6.8	85.1 ± 6.9	<0.01	88.0 ± 6.7	79.8 ± 7.2	<0.01
Systolic blood pressure, mmHg	124.5 ± 13.3	131.3 ± 11.7	125.4 ± 11.5	<0.01	131.1 ± 13.5	117.9 ± 13.3	<0.01
Diastolic blood pressure, mmHg	78.1 ± 9.4	82.3 ± 8.8	78.9 ± 8.6	<0.01	80.8 ± 9.4	74.2 ± 9.3	<0.01
Comorbidity (%)	3436 (42.5)	913 (70.4)	1331 (37.6)	<0.01	521 (70.7)	671 (26.7)	<0.01
Hypertension	2248 (27.8)	674 (52.0)	830 (23.5)	<0.01	395 (53.6)	349 (13.9)	<0.01
Diabetes	923 (11.4)	386 (29.8)	279 (7.9)	<0.01	202 (27.4)	56 (2.2)	<0.01
Dyslipidemia	1023 (12.7)	242 (18.7)	328 (9.3)	<0.01	155 (21.0)	298 (11.9)	<0.01
Cardiovascular disease	196 (2.4)	60 (4.6)	95 (2.7)	0.001	13 (1.8)	28 (1.1)	0.188
Smoking status (%)				<0.01			0.944
Never smoker	4154 (52.0)	210 (16.5)	838 (24.1)		704 (95.9)	2402 (96.0)	
Ex-smoker	2130 (26.7)	545 (42.8)	1523 (43.8)		15 (2.0)	47 (1.9)	
Current smoker	1705 (21.3)	519 (40.7)	1117 (32.1)		15 (2.0)	54 (2.2)	
Alcohol consumption				<0.01			<0.01
Never	3202 (39.6)	262 (20.2)	740 (20.9)		570 (77.3)	1630 (65.0)	
Moderate	2622 (32.5)	419 (32.3)	1425 (40.3)		120 (16.3)	658 (26.2)	
Heavy	2256 (27.9)	615 (47.5)	1374 (38.8)		47 (6.4)	220 (8.8)	
Physical activity (%)				<0.01			0.051
Sedentary	3065 (37.9)	514 (39.7)	1106 (31.3)		357 (48.4)	1088 (43.4)	
Light	2463 (30.5)	427 (32.9)	1179 (33.3)		180 (24.4)	677 (27.0)	
Moderate-to-vigorous	2553 (31.6)	355 (27.4)	1254 (35.4)		200 (27.1)	744 (29.7)	
Fasting blood glucose, mg/dL	95.0 ± 22.5	111.4 ± 30.7	93.0 ± 19.6	<0.01	105.0 ± 26.4	86.5 ± 12.6	<0.01
Triglyceride, mg/dL	110.5 ± 73.9	186.2 ± 102.7	101.2 ± 56.2	<0.01	86.0 ± 3.2	77.6 ± 34.6	<0.01
High-density lipoprotein, mg/dL	53.9 ± 15.9	41.3 ± 11.2	52.5 ± 13.4	<0.01	48.5 ± 13.0	64.0 ± 15.7	<0.01

BMI, body mass index; MetS, metabolic syndrome. Data are presented as mean ± standard deviation or numbers (percentages); *p*-values were calculated using Pearson’s chi-squared test for categorical variables and Student’s *t*-test for numerical variables.

**Table 2:** Computed tomography findings of the study participants (*n* = 8081).

Variables	Total (*n* = 8081)	Male (*n* = 4835)			Female (*n* = 3246)			*p*-Value *
		MetS (*n* = 1296)	No MetS (*n* = 3539)	*p*-Value	MetS (*n* = 737)	No MetS (*n* = 2509)	*p*-Value	
Subcutaneous fat area, cm^2^	143.7 ± 60.1	158.6 ± 62.0	124.8 ± 53.9	<0.01	188.6 ± 59.6	149.6 ± 57.2	<0.01	<0.01
Visceral fat area, cm^2^	104.8 ± 61.4	171.2 ± 57.6	111.6 ± 54.6	<0.01	110.4 ± 43.9	59.3 ± 35.2	<0.01	<0.01
Extramyocellular lipids area, cm^2^	5.5 ± 4.0	6.7 ± 4.4	5.0 ± 3.5	<0.01	7.2 ± 4.6	5.2 ± 4.0	<0.01	0.023
Total abdominal muscle area, cm^2^	137.0 ± 34.4	168.0 ± 24.1	156.9 ± 22.3	<0.01	107.1 ± 15.2	101.7 ± 13.2	<0.01	<0.01
Normal-attenuation muscle area, cm^2^	112.1 ± 32.0	135.0 ± 24.0	132.0 ± 21.9	<0.01	80.0 ± 16.4	81.6 ± 13.8	0.009	<0.01
Low-attenuation muscle area, cm^2^	24.9 ± 10.5	33.1 ± 11.7	24.9 ± 9.8	<0.01	27.0 ± 9.6	20.0 ± 7.6		
Adipose tissue index, cm^2^/(kg/m^2^)								
Subcutaneous fat area index	5.9 ± 2.0	5.9 ± 1.8	5.1 ± 1.8	<0.01	7.4 ± 1.9	6.6 ± 2.0	<0.01	<0.01
Visceral fat area index	4.2 ± 2.2	6.4 ± 1.9	4.6 ± 2.0	<0.01	4.3 ± 1.5	2.6 ± 1.3	<0.01	<0.01
Extramyocellular lipids area index	0.15 ± 0.22	0.25 ± 0.16	0.21 ± 0.13	<0.01	0.28 ± 0.17	0.23 ± 0.16	<0.01	<0.01
Skeletal muscle index, cm^2^/(kg/m^2^)								
Total abdominal muscle area index	5.7 ± 1.2	6.4 ± 0.7	6.6 ± 0.7	<0.01	4.2 ± 0.5	4.6 ± 0.6	<0.01	<0.01
Normal-attenuation muscle index	4.7 ± 1.2	5.1 ± 0.8	5.5 ± 0.9	<0.01	3.2 ± 0.7	3.7 ± 0.7	<0.01	<0.01
Low-attenuation muscle area index	1.0 ± 0.4	1.2 ± 0.4	1.0 ± 0.4	<0.01	1.1 ± 0.3	0.9 ± 0.3	<0.01	<0.01
The ratio of low-attenuation muscle area to total abdominal muscle area, %	18.7 ± 7.6	19.8 ± 7.0	15.9 ± 7.0	<0.01	25.6 ± 9.3	19.9 ± 7.5	<0.01	<0.01

MetS, metabolic syndrome. Total abdominal muscle area was calculated by summing the normal-attenuation muscle area and low-attenuation muscle area. Data are presented as mean ± standard deviation. * *p*-values were calculated for differences between males and females with metabolic syndrome. All *p*-values were calculated using Student’s *t*-test.

**Table 3:** Pearson’s correlation coefficients of metabolic risk factors and the TAMA, NAMA, LAMA, SFA, VFA, and EMCLA indices in males and females.

Variables	Male						Female					
	TAMA Index	NAMA Index	LAMA Index	SFA Index	VFA Index	EMCLA Index	TAMA Index	NAMA Index	LAMA Index	SFA Index	VFA Index	EMCLA Index
Waist circumference	−0.205	−0.360	0.441	0.323	0.601	0.323	−0.455	−0.545	0.406	0.358	0.608	0.358
Log triglycerides	−0.089	−0.135	0.141	0.095	0.376	0.095	−0.189	−0.216	0.144	0.119	0.422	0.119
Log HDL cholesterol	0.089	0.136	−0.144	−0.060	−0.272	−0.060	0.189	0.211	−0.133	−0.071	−0.373	−0.071
Log fasting blood glucose	−0.117	−0.160	0.147	0.065	0.238	0.065	−0.198	−0.250	0.209	0.143	0.388	0.143
Diastolic blood pressure	−0.053	−0.099	0.128	0.074	0.154	0.074	−0.187	−0.221	0.158	0.140	0.264	0.140
Systolic blood pressure	−0.076	−0.125	0.144	0.100	0.153	0.100	−0.246	−0.303	0.241	0.189	0.348	0.189

All *p*-values are <0.01. TAMA, total abdominal muscle area; NAMA, normal-attenuation muscle area; LAMA, low-attenuation abdominal muscle area; SFA, subcutaneous fat area; VFA, visceral fat area; EMCLA, extramyocellular lipids area; HDL, high-density lipoprotein.

**Table 4:** Multivariate analyses between abdominal muscle index and metabolic syndrome.

Variables	Male		Female			
	(*n* = 4835)		Postmenopause (*n* = 2197)		Premenopause (*n* = 1049)	
	OR (95% CI)	*p*-Value	OR (95% CI)	*p*-Value	OR (95% CI)	*p*-Value
Total abdominal muscle area index, cm^2^/(kg/m^2^)	0.937 (0.835–1.052)	0.271	0.820 (0.658–1.022)	0.077	0.546 (0.352–0.941)	0.028
Normal-attenuation muscle index, cm^2^/(kg/m^2^)	0.836 (0.743–0.940)	0.003	0.754 (0.601–0.946)	0.015	0.534 (0.321–0.887)	0.015
Low-attenuation muscle index, cm^2^/(kg/m^2^)	1.771 (1.359–2.308)	<0.01	1.402 (0.861–2.284)	0.174	1.420 (0.350–5.765)	0.623
The ratio of low-attenuation muscle area to total abdominal muscle area, %	1.040 (1.023–1.058)	<0.01	1.025 (1.004–1.046)	0.022	1.050 (0.987–1.117)	0.124

CI, confidence interval; OR, odds ratio. Adjusted for age, comorbidities (e.g., hypertension, diabetes, dyslipidemia, cardiovascular disease), smoking status, alcohol consumption, physical activity, visceral fat index, and extramyocellular fat area index.

## Appendix A

**Table A1:** Multivariate analyses between abdominal muscle index and metabolic syndrome after excluding participants with comorbidities.

Variables	Male		Female			
	(*n* = 2591)		Postmenopause (*n* = 1164)		Premenopause (*n* = 890)	
	OR (95% CI)	*p*-Value	OR (95% CI)	*p*-Value	OR (95% CI)	*p*-Value
Total abdominal muscle area index, cm^2^/(kg/m^2^)	0.836	0.056	0.817	0.285	0.628	0.179
	(0.696–1.005)		(0.563–1.184)		(0.319–1.238)	
Normal-attenuation muscle	0.784	0.011	0.863	0.447	0.636	0.194
index, cm^2^/(kg/m^2^)	(0.651–0.946)		(0.590–1.262)		(0.321–1.259)	
Low-attenuation muscle index, cm^2^/(kg/m^2^)	1.402	0.142	0.810	0.797	0.868	0.887
	(0.894–2.199)		(0.163–4.031)		(0.123–6.144)	
The ratio of low-attenuation	1.032	0.034	0.994	0.767	1.014	0.737
muscle area to total abdominal muscle area, %	(1.002–1.063)		(0.956–1.034)		(0.933–1.103)	

CI, confidence interval; OR, odds ratio. Adjusted for age, smoking status, alcohol consumption, physical activity, visceral fat index, and extramyocellular fat area index.
